# Data concerning isometric lower limb strength of dominant *versus* not-dominant leg in young elite soccer players

**DOI:** 10.1016/j.dib.2018.01.022

**Published:** 2018-01-31

**Authors:** Mehdi Rouissi, Moktar Chtara, Nicola Luigi Bragazzi, Monoem Haddad, Karim Chamari

**Affiliations:** a Tunisian Research Laboratory "Sport Performance Optimization", National Center of Medicine and Science in Sports Tunisia; b Department of Mathematics (DIMA), University of Genoa, Genoa, Italy; c School of Public Health, Department of Health Sciences (DISSAL), University of Genoa, Genoa, Italy; d Sport Science Program, College of Arts and Sciences, Qatar University, Doha, Qatar; e Athlete Health and Performance Aspetar, Qatar Orthopaedic and Sports Medicine Hospital, Doha, Qatar

**Keywords:** Lower limb strength, leg, Not-dominant leg, Handheld dynamometer, Soccer players

## Abstract

The present data article describes the isometric lower limb strength of dominant leg *versus* not-dominant leg measured with handheld dynamometer (HHD) in a sample of 31 young elite soccer players (age 16.42 ± 0.45 years; height 169.00 ± 0.50 cm; leg length 94.80 ± 3.32 cm; body-mass 67.04 ± 5.17 kg).

**Specifications Table**

**Table:** 

Subject area	*Sports sciences*
More specific subject area	*Sports data mining*
Type of data	*Tables and graphs*
How data was acquired	*Isometric strength test administered to a sample of 31 athletes*
Data format	*Raw and Analyzed*
Experimental factors	*Data were obtained using a handheld dynamometer*
Experimental features	*Reliability coefficients, paired Student's t-test*
Data source location	*Tunisia*
Data accessibility	Data are within this article

**Value of the data**

• These data could be further statistically refined, processed and eventually integrated with other data to build a mathematical predictive model concerning isometric lower limb strength of dominant *versus* not-dominant leg measured with handheld dynamometer (HHD).
• These data could be useful for sports managers, coaches, scientists and athletes for designing and implementing *ad hoc* training programs and interventions.

## 1 Data

**Fig. 1:**
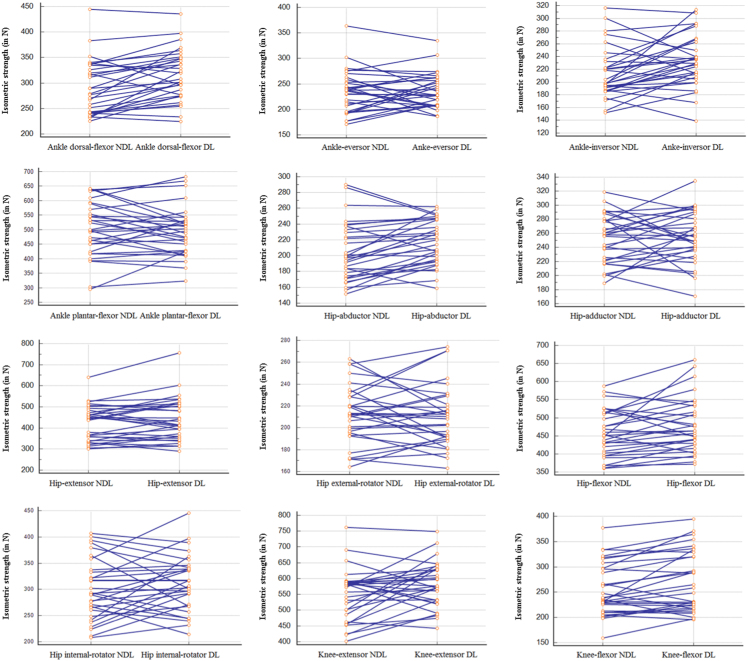
Isometric strength (in N) of the dominant leg (DL) *versus* not-dominant leg (NDL).

**Fig. 2:**
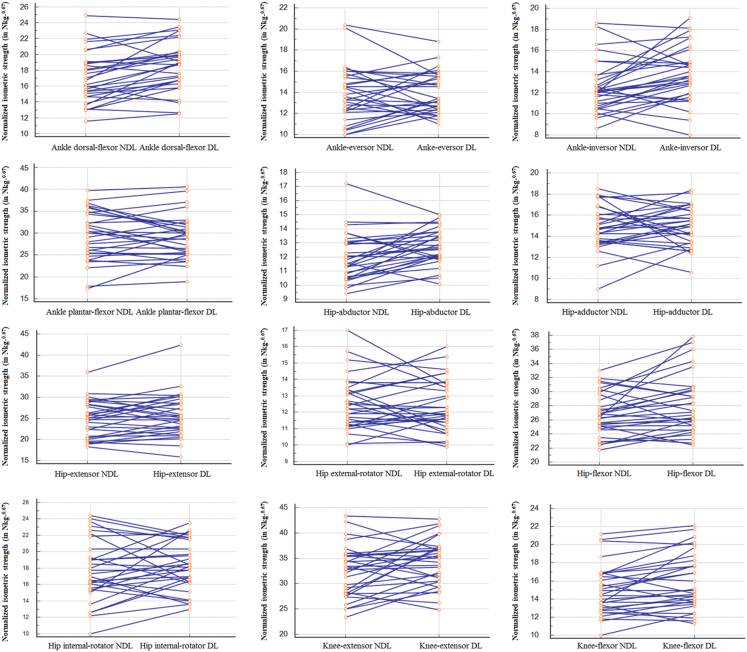
Normalized isometric strength (in N kg-^0.67^) of the DL *versus* NDL.

This paper contains data concerning allometric test administered to a sample of 31 male athletes from north Africa (Tunisia), with at least 6 years of soccer practice, measured with a handheld dynamometer (Microfet 2, Hoggan Health Industries, Inc., Draper, UT) [1]. General characteristics of the sample are reported in Table 1. The impact of dominant *versus* not-dominant leg on the allometric test is shown in Table 2 and in Fig. 1 and, after body-mass normalization, in Table 3 and in Fig. 2. Table 4 reports the reliability coefficients of the allometric test. Each muscle group was examined twice for reliability.

**Table 1:** General characteristics of the recruited sample.

**Variable**	**Mean**	**SD**
Age (years)	16.42	0.45
Height (cm)	169.00	0.50
Leg length (cm)	94.80	3.32
Body-mass (kg)	67.04	5.17

SD: standard deviation.

**Table 2:** Results of paired Student's *t*-test comparing isometric strength of the dominant *versus* not-dominant leg.

**Muscle**	**Dominant leg**		**Not-dominant leg**		**Sig.**
	**Mean**	**SD**	**Mean**	**SD**	
Hip-abductor	217.31	28.35	205.08	36.58	0.0069
Hip-adductor	255.19	36.08	251.33	34.25	0.5502
Hip-flexor	478.67	75.41	456.92	64.15	0.0282
Hip-extensor	439.59	101.06	423.98	83.50	0.0937
Hip internal-rotator	310.98	53.10	300.74	57.55	0.2862
Hip external-rotator	210.99	28.35	212.43	26.42	0.7343
Knee-flexor	271.79	60.03	255.64	51.14	0.0042
Knee-extensor	580.64	70.86	549.89	80.81	0.0313
Ankle plantar-flexor	493.79	84.55	499.06	93.46	0.6395
Ankle dorsal-flexor	315.01	49.08	290.63	52.85	0.0004
Ankle-inversor	233.01	40.35	212.99	40.08	0.0073
Ankle-eversor	236.92	33.96	234.79	41.35	0.7409

Sig: statistical significance.

**Table 3:** Results of paired Student's *t*-test comparing isometric strength of the dominant *versus* non-dominant leg, after body-mass normalization.

**Muscle**	**Dominant leg**		**Not-dominant leg**		**Sig.**
	**Mean**	**SD**	**Mean**	**SD**	
Hip-abductor	12.75	1.25	12.00	1.67	0.0053
Hip-adductor	14.97	1.87	14.80	2.09	0.6589
Hip-flexor	28.13	4.14	26.79	3.07	0.0208
Hip-extensor	25.70	5.05	24.84	4.33	0.1115
Hip internal-rotator	18.28	3.00	17.77	3.73	0.3625
Hip external-rotator	12.40	1.58	12.50	1.61	0.6801
Knee-flexor	15.92	3.13	14.99	2.74	0.0055
Knee-extensor	34.22	4.59	32.40	5.07	0.0304
Ankle plantar-flexor	29.05	4.97	29.41	5.74	0.5857
Ankle dorsal-flexor	18.57	3.10	17.13	3.20	0.0004
Ankle-inversor	13.75	2.61	12.54	2.41	0.0062
Ankle-eversor	13.94	1.94	13.85	2.57	0.8104

Sig: statistical significance.

**Table 4:** Reliability results of the isometric strength tests.

Muscle		ICC_s_	(95%CI)	SEM	CV%
Hip-abductor	DL	Excellent	(0.94–0.97)	5.22	5.36
	NDL	Good	(0.74–0.81)	7.36	5.45
Hip-adductor	DL	Excellent	(0.90–0.94)	6.47	6.48
	NDL	Excellent	(0.84–0.87)	4.84	5.87
Hip-flexor	DL	Excellent	(0.91–0.95)	8.91	7.55
	NDL	Excellent	(0.92–0.96)	6.37	5.39
Hip-extensor	DL	Excellent	(0.84–0.89)	8.66	8.78
	NDL	Excellent	(0.88–0.90)	7.45	6.22
Hip internal-rotator	DL	Excellent	(0.90–0.93)	9.34	7.64
	NDL	Good	(0.75–0.82)	6.71	5.67
Hip external-rotator	DL	Excellent	(0.87–0.91)	8.38	8.72
	NDL	Excellent	(0.93–0.95)	9.75	5.69
Knee-flexor	DL	Good	(0.72–0.80)	11.39	6.71
	NDL	Excellent	(0.89–0.92)	8.78	5.24
Knee-extensor	DL	Excellent	(0.76–0.84)	9.33	7.78
	NDL	Excellent	(0.85–0.92)	12.74	8.48
Ankle plantar-flexor	DL	Excellent	(0.90–0.95)	8.97	9.46
	NDL	Excellent	(0.77–0.82)	6.44	5.94
Ankle dorsal-flexor	DL	Excellent	(0.79–0.84)	14.88	8.45
	NDL	Excellent	(0.94–0.97)	11.37	6.36
Ankle-inversor	DL	Excellent	(0.93–0.96)	7.30	7.42
	NDL	Excellent	(0.86–0.90)	5.64	5.59
Ankle-eversor	DL	Excellent	(0.91–0.93)	6.89	8.37
	NDL	Good	(0.73–0.85)	7.24	6.64

CI: confidence Interval; CV: coefficient of variation; DL: dominant leg; NDL: not-dominant leg ICCs: intraclass correlation coefficients; SEM: standard error of measurement.

## 2 Experimental design, materials and methods

Intraclass correlation coefficients (ICCs) were used to quantitatively assess the test-retest reliability of muscle strength measurement with HHD. Also Standard Error of Measurement (SEM) and coefficient of variation (CV) were computed.

All statistical analyses were performed using the commercial software Statistical Package for Social Science (SPSS, version 23.0, IL, USA) and MedCalc Statistical Software version 16.8.4 (MedCalc Software bvba, Ostend, Belgium; https://www.medcalc.org; 2016). Figures with a *p*-value < 0.05 were considered statistically significant.
